# pH sensing and inflammation: linking acidic microenvironments to immune reprogramming

**DOI:** 10.3389/fimmu.2026.1879529

**Published:** 2026-07-27

**Authors:** Mingyu Zhang, Jiaju Tian, Weiming Sun

**Affiliations:** 1 The First Clinical Medical College, Lanzhou University, Lanzhou, Gansu, China; 2 The First Hospital of Lanzhou University, Endocrinology Department, Lanzhou, Gansu, China

**Keywords:** acidic microenvironment, acid-sensing ion channels, BRD4, immune reprogramming, inflammation, liquid–liquid phase separation, pH sensing, proton-sensing GPCRs

## Abstract

As a critical variable of physiological homeostasis, pH has traditionally been regarded as a metabolic byproduct. However, accumulating evidence indicates that pH also functions as an active signaling cue capable of modulating the initiation and regulation of inflammation via proton-sensing receptors. Tissue acidification is widely observed across pathological conditions, including infection, ischemia, cancer, and chronic inflammatory diseases. Acidic microenvironments not only influence immune cell metabolism and effector functions but also promote the release of inflammatory mediators and contribute to tissue injury.

The immune system harbors a diverse array of proton sensors, including proton-sensitive G protein-coupled receptors (GPCRs; e.g., GPR4, GPR65, GPR68), acid-sensing ion channels (ASICs), transient receptor potential (TRP) channels, intracellular pH sensors and pH-responsive regulatory factors, including BRD4, HIF-1α, SMAD5, and SREBP2. These sensors transduce local pH fluctuations into specific signaling cascades and gene expression programs, thereby reshaping inflammatory responses.

In this review, we systematically summarize recent advances in understanding pH sensing in the regulation of inflammation. We focus on (i) the structural classes and distribution patterns of pH sensors, (ii) the molecular integration of acidic signals within inflammatory signaling networks and their transcriptional regulatory mechanisms, and (iii) the roles of pH sensing in immune homeostasis and pathological inflammation. Finally, we highlight emerging opportunities for identifying pH-based diagnostic biomarkers and developing targeted therapeutic interventions. Collectively, this work underscores the conceptual and translational significance of the “pH–sensor–inflammation” axis as an emerging paradigm in immune regulation.

## 1 Introduction

pH is a fundamental physicochemical parameter essential for maintaining cellular and tissue homeostasis. Even subtle fluctuations in pH can profoundly influence metabolic reactions, signal transduction pathways, and gene regulatory networks. Under physiological conditions, acid–base balance is tightly regulated by the bicarbonate buffering system, proton pumps, and diverse ion transporters, thereby ensuring an optimal biochemical environment for cellular processes (1, 2). However, in the context of infection, ischemia, or metabolic stress, this homeostatic equilibrium is frequently disrupted, leading to localized tissue acidification as a common pathological feature. Traditionally, local acidosis has been regarded as a simple byproduct of enhanced metabolic activity, such as lactate accumulation derived from glycolysis. In recent years, however, emerging evidence has demonstrated that pH is not merely a passive indicator of metabolic status but also an actively sensed signaling input capable of modulating cell fate decisions and immune phenotypes through proton-sensitive molecular mechanisms (3–5). Accordingly, pH has evolved from being viewed solely as a biochemical parameter to being recognized as a critical integrative node linking cellular metabolism, signaling networks, and functional adaptation.

Disruption of acid–base homeostasis is closely associated with a broad spectrum of pathological conditions. During ischemia–reperfusion (I/R) injury, extracellular acidification frequently occurs and can potentiate inflammatory signaling cascades (6). In chronic inflammatory states, heightened glycolytic activity in immune cells commonly leads to lactate accumulation and sustained local acidosis. Within the tumor microenvironment, extracellular acidification driven by the Warburg effect not only remodels the extracellular matrix but also suppresses effector T cell function, thereby fostering an immunosuppressive niche (7–9). Collectively, these observations indicate that acidic microenvironments are not merely byproducts of disease progression but represent critical determinants of inflammatory dynamics. Local pH fluctuations can reshape immune cell metabolism and function by altering membrane potential, ion permeability, and the activity of intracellular signaling pathways. In this context, acidification should be regarded as a central regulatory signal within inflammatory networks, capable of actively modulating both the magnitude and direction of immune responses.

Inflammation constitutes a fundamental host defense response to infection, tissue injury, and metabolic imbalance, aiming to eliminate harmful stimuli and promote tissue repair (10). Under physiological conditions, the initiation and resolution of inflammation are tightly regulated to prevent excessive immune activation (11–14). However, when the intensity or duration of inflammatory responses becomes dysregulated, inflammation may transition into a chronic pathological process, contributing to the onset and progression of diseases such as rheumatoid arthritis, inflammatory bowel disease, atherosclerosis, and cancer (15, 16). Traditional studies have primarily focused on canonical inflammatory mediators, including cytokines, chemokines, reactive oxygen species (ROS), and damage-associated molecular patterns (DAMPs). Increasingly, however, it is recognized that inflammatory responses do not occur in isolation from the physicochemical context of the tissue microenvironment. Rather, inflammation represents a dynamic system intricately coupled with tissue metabolism, redox status, and acid–base fluctuations (17–19). Within this conceptual framework, pH—serving as an integrative signal reflecting bidirectional interactions between metabolism and inflammation—is emerging as a critical frontier in immunological research.

Accumulating evidence demonstrates that the immune system harbors diverse proton-sensing mechanisms. Tissues and cells are capable of detecting pH fluctuations through specialized pH sensors and translating these changes into defined signaling networks and gene expression programs, thereby modulating inflammatory responses. For instance, proton-sensitive G protein–coupled receptors (GPCRs), including GPR4, GPR65, and GPR68, are activated under acidic conditions and regulate immune cell migration and cytokine secretion (3). Acid-sensing ion channels (ASICs) influence neuronal and immune cell functions through modulation of calcium signaling (4). Transient receptor potential (TRP) channels act as polymodal sensors that couple pH alterations to inflammatory signaling pathways (20). In addition, intracellular pH sensors and pH-responsive regulatory factors such as HIF-1α and BRD4 have been shown to exhibit pH-dependent regulatory activity (21–23), highlighting a pivotal role for acid–base signals in gene reprogramming.

In this review, we systematically summarize recent advances in understanding the interplay between pH sensing and inflammatory regulation. Beginning with proton detection at the level of membrane receptors (GPCRs, ASICs, and TRPs), we extend the discussion to nuclear factors—particularly BRD4—and their phase separation behavior and epigenetic regulatory functions under acidic conditions. We aim to elucidate how acid–base signals traverse cellular membranes and chromatin landscapes to coordinately shape immunometabolic states and transcriptional reprogramming. Special emphasis is placed on the structural features, signaling coupling properties, and network integration patterns of distinct classes of pH sensors, as well as their functional roles in homeostatic maintenance, pathological inflammation, and tumor immunity. By integrating membrane-level sensing with nuclear regulatory mechanisms, this review seeks to define the conceptual framework of the “pH–sensor–inflammation” axis as an emerging paradigm in immune regulation and to provide new theoretical foundations for the diagnosis and therapeutic targeting of diseases associated with acidic microenvironments.

## 2 Structural characteristics of pH sensors

**Figure 1:**
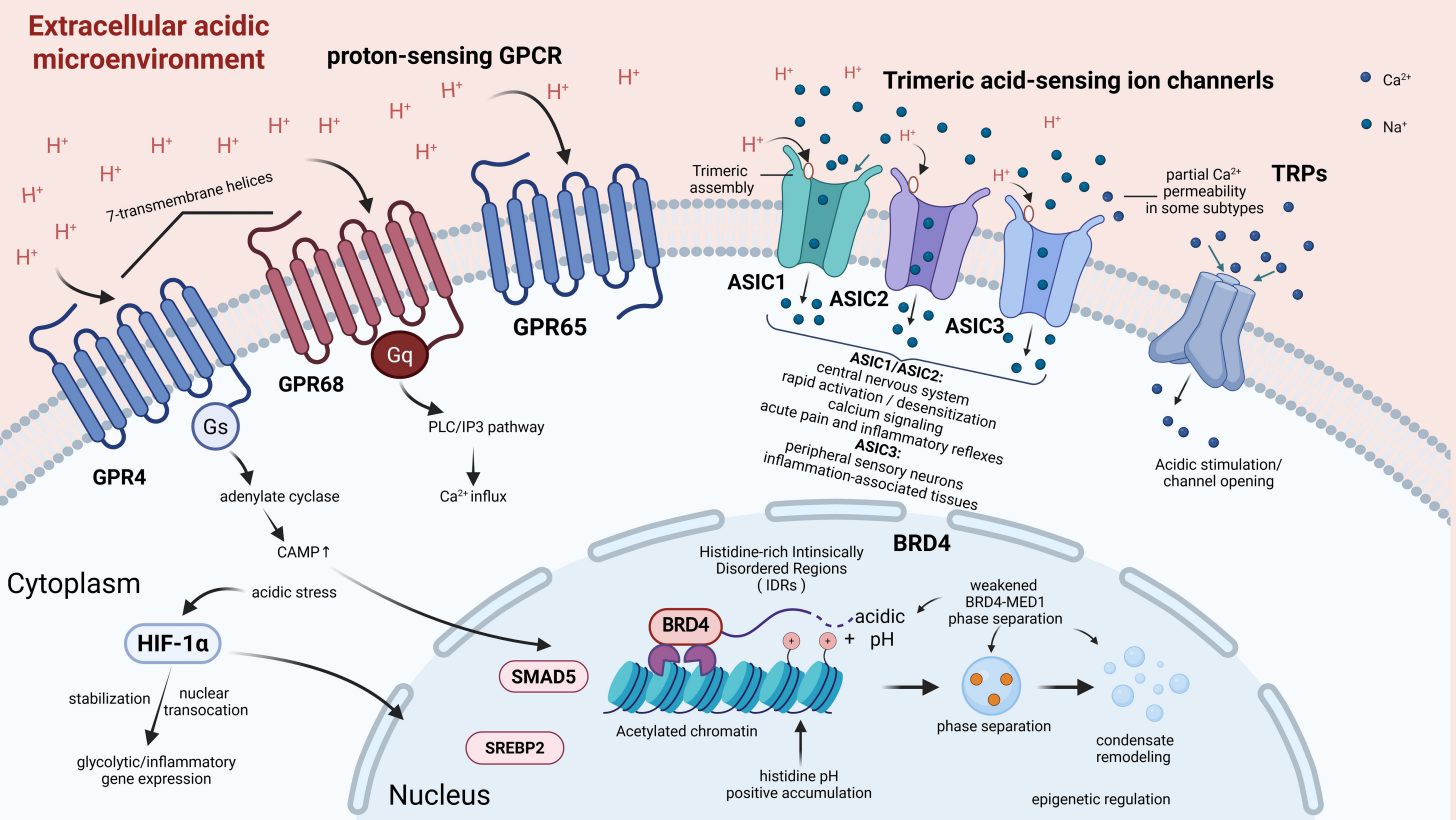
Structural characteristics of pH sensors.

The ability of the organism to detect acid–base fluctuations relies on a multilayered molecular system, encompassing membrane-localized receptors and ion channels as well as intracellular transcriptional regulators. Membrane-level sensors directly perceive extracellular acidification signals, whereas intracellular factors reflect metabolic status and nuclear homeostasis. Together, these components constitute an integrated proton-sensing network that enables coordinated cellular responses to pH dynamics (**Figure 1**).

### 2.1 Membrane-associated pH sensors

Proton-sensing GPCRs, such as GPR4, GPR65, and GPR68, are typical seven-transmembrane receptors, and their proton sensing does not rely solely on the protonation of extracellular histidines, but is mediated by a cooperative network jointly formed by titratable residues such as histidine, aspartic acid, and glutamic acid (24, 25). Different receptors may show different G protein-coupling bias under acidic conditions, among which GPR4 and GPR65 mainly signal through the Gs-cAMP pathway, whereas GPR68 may exhibit dual Gq/Gs coupling characteristics, and GPR132 is also associated with acidic or lipid metabolism-abnormal inflammatory microenvironments, but its direct proton-sensing role is still less clear than that of GPR4, GPR65, and GPR68 (26–33). These differences confer distinct signaling outputs in various tissues and immune cell types. GPR4 is highly expressed in vascular endothelial cells and participates in the regulation of vascular inflammation (32, 34); GPR65 is enriched in lymphocytes and macrophages, with its expression and function significantly altered under inflammatory conditions (35); GPR68 (OGR1) is present in fibroblasts and is upregulated during inflammatory and fibrotic conditions, while also functioning in certain immune cells (36); GPR132 is mainly found in macrophages and tumor-associated cells (37, 38).

Acid-sensing ion channels (ASICs) represent another important class of membrane-associated sensors. They belong to the DEG/ENaC family and typically assemble as functional trimers, which can be either homotrimeric or heterotrimeric complexes, with subunit composition determining channel kinetics and pH sensitivity. Different ASIC subtypes exhibit distinct thresholds for acid activation (pH_50_ ranging approximately from 4.5 to 6.8) (39–43). Upon activation, ASICs primarily mediate Na^+^ influx (with certain subtypes also permeable to Ca²^+^), rapidly converting acidic stimuli into changes in membrane potential and calcium signaling (44, 45). The rapid activation and desensitization kinetics of ASICs enable them to effectively capture transient acidic insults, rendering them well suited as “triggers” of acute pain and inflammatory reflexes (41, 42). ASIC1 and ASIC2 are abundantly expressed in the central nervous system, whereas ASIC3 is predominantly distributed in peripheral sensory neurons and inflammation-associated tissues, where it contributes to acid-induced nociception and acute inflammatory responses (46, 47).

Increasing evidence suggests that ASICs may also directly participate in inflammatory regulation within immune cells. Expression of ASICs has been detected in macrophages, dendritic cells, and other immune cell populations, and their activation may influence the production of inflammatory mediators, although the overall evidence remains to be further substantiated. For example, recent studies indicate that ASIC1a signaling can drive macrophage polarization toward a pro-inflammatory M1 phenotype, thereby exacerbating joint inflammation and tissue damage (45).

Transient receptor potential (TRP) channels provide a multimodal platform for acid–base sensing. Subtypes such as TRPV1 and TRPA1 are highly sensitive to acidic stimuli (48, 49). Structurally, TRP channels are tetramers, with each subunit containing six transmembrane helices (S1–S6), and the S5–S6 segments forming the central pore and selectivity filter (50–52). Acidification can alter channel gating mechanisms, promoting channel opening and inducing rapid Ca²^+^ influx (49, 53). Unlike ASICs, TRP channels are often simultaneously sensitive to temperature, mechanical forces, and chemical ligands, positioning them as key integrators of multiple stimuli (50, 51). In terms of distribution, TRPV1 is widely expressed in sensory nerve endings as well as in respiratory and gastrointestinal epithelia, whereas TRPA1 is prominently expressed in sensory neurons of the respiratory tract and skin (54). Transcripts or proteins of certain TRP family members have also been detected in immune cells such as dendritic cells and mast cells, with reported effects on cell migration and pro-inflammatory mediator release (55, 56). Compared with ASICs, TRP channels are more likely to function under conditions of chronic acidification, integrating nociceptive signaling with inflammatory pathways.

Although different membrane pH sensors respond to acidic microenvironments, their structural bases and activation mechanisms are not the same. Proton-sensing GPCRs mainly rely on extracellular histidine and carboxylate residue networks as pH-sensing motifs; during acidification, histidine protonation can induce conformational rearrangements of extracellular loops and transmembrane helices, and further alter the intracellular G protein-coupling interface, thereby initiating second messenger pathways such as cAMP and Ca²^+^ (24, 25). In contrast, the acid-sensitive sites of ASICs are mainly concentrated in “acidic pockets” enriched in aspartic acid and glutamic acid at the interfaces of trimeric subunits; Protonation of these pockets induces relative movements of extracellular domains and couples these conformational changes to transmembrane pore opening, rapidly converting acidic stimuli into Na^+^ influx and membrane depolarization (39–45). TRP channels are characterized by multimodal integration; their acid sensitivity involves the pore region, extracellular loops, and intracellular regulatory domains, allowing them to integrate signals from acid, heat, chemical stimuli, and inflammatory mediators simultaneously. Therefore, in many inflammatory contexts, GPCRs preferentially mediate sustained second messenger regulation, ASICs are more suitable for sensing rapid acidification shocks, whereas TRPs often connect acidification, pain sensation, and neurogenic inflammation.

### 2.2 Intracellular pH sensors and pH-responsive regulatory factors

**Figure 2:**
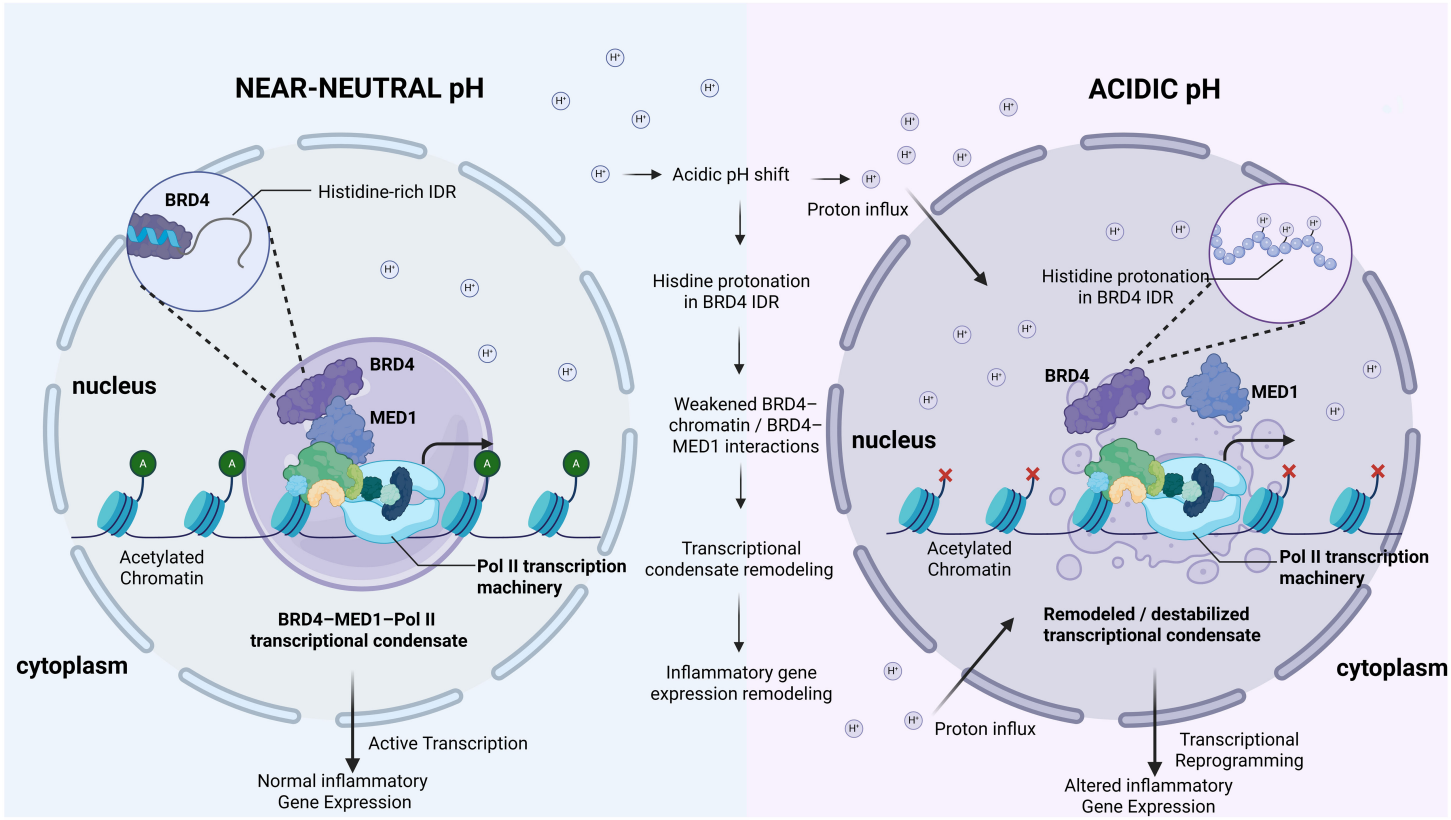
BRD4-mediated intracellular pH sensing and transcriptional condensate remodeling.

Unlike membrane receptors and ion channels that directly sense extracellular pH, intracellular acid–base changes can further influence transcriptional, metabolic, and epigenetic programs through pH sensors and various pH-responsive regulatory factors. Among them, BRD4 has recently been identified as an intracellular pH sensor, providing a new mechanistic perspective for understanding how acidification directly regulates inflammatory transcription (22). BRD4 contains tandem bromodomains and can bind acetylated chromatin; under acidic conditions, its histidine-rich intrinsically disordered region can respond to a decrease in intracellular pH, altering the stability of BRD4/MED1 transcriptional condensates and its chromatin-binding state,thereby reshaping the expression of inflammation-related genes in a locus-specific manner (22). This mechanism is based on the ability of coactivators such as BRD4/MED1 to organize super-enhancers and the transcriptional machinery through liquid–liquid phase separation (57), and is also consistent with the physical mechanism by which BRD4 condensates are regulated by binding to acetylated chromatin (58). Therefore, liquid–liquid phase separation is not only an organizational mode of transcriptional regulation, but may also constitute a noncanonical mechanism of intracellular pH sensing and signal transduction. However, BRD4-mediated pH sensing should not be reduced to a unidirectional pro-inflammatory or anti-inflammatory effect, but should be understood as an epigenetic calibration mechanism dependent on cell type, chromatin context, and the degree of acidification (**Figure 2**).

Beyond BRD4, HIF-1α, SMAD5, and SREBP2 represent additional intracellular regulators that link acid–base status to inflammatory metabolism and gene expression. Although these molecules are not all direct pH sensors in the strict sense, they provide important routes through which intracellular pH fluctuations can be integrated into cellular regulatory programs. HIF-1α is classically recognized as an oxygen-regulated transcription factor, but its stability and transcriptional activity are also shaped by acidification and metabolic context. In certain tumor contexts, acidification can promote HIF function through HSP90-related mechanisms (59), whereas under specific conditions in which acidification and hypoxia coexist, acidification may also weaken the hypoxic stabilization of HIF-1α by enhancing lysosomal degradation (60), suggesting that the response of HIF-1α to acidification is strongly dependent on cell type and microenvironment. SMAD5 provides a representative example linking intracellular pH to bioenergetic homeostasis. In addition to participating in canonical BMP/TGF-β-related transcriptional pathways, the noncanonical cytoplasmic functions of SMAD5 can also respond to changes in intracellular pH. Changes in intracellular pH can affect the subcellular localization of SMAD5 and its interactions with metabolic enzymes, enabling its participation in the regulation of glycolysis and energy homeostasis (61). SREBP2 reflects the impact of acidification on lipid metabolism and remodeling of the inflammatory microenvironment. Low-pH conditions can promote the nuclear translocation of SREBP2 and the expression of cholesterol biosynthesis-related genes, thereby enhancing the mevalonate pathway and cholesterol metabolic reprogramming (62). In addition, SREBP2 itself can promote the transcriptional activation of lipogenesis-related genes and the regulation of cholesterol homeostasis through nuclear condensates (63). Because cholesterol metabolism directly affects membrane structure, lipid raft signaling, inflammatory receptor clustering, and immune cell function, SREBP2 can be regarded as an important intracellular node connecting acidic microenvironments, lipid metabolism, and inflammatory responses.

Overall, BRD4-mediated remodeling of pH-dependent transcriptional condensates provides a direct mechanistic link between intracellular acid–base sensing and epigenetic regulation. By comparison, HIF-1α, SMAD5, and SREBP2 function primarily as pH-responsive regulatory nodes that integrate acidic signals into hypoxia-associated metabolism, bioenergetic control, and cholesterol metabolism, respectively. Together, these mechanisms suggest that intracellular pH changes can be translated into sustained metabolic adaptation and inflammatory transcriptional remodeling, rather than remaining as transient physicochemical disturbances.

### 2.3 Evolutionary perspective of pH sensing

Cross-species comparisons further illuminate the evolutionary characteristics of pH sensors. Phylogenetic and functional studies indicate that ASIC and TRP families emerged in non-mammalian vertebrates and even earlier lineages, supporting the notion that acid–base sensing represents an ancient and evolutionarily conserved molecular mechanism (64, 65). In contrast, proton-sensing GPCRs have evolved more complex distribution patterns and immune-related functions in mammals. This receptor family was first described by Ludwig et al. (66) and was subsequently shown in tumor and inflammatory models to exert physiological and pathological effects in acidic microenvironments (67–69). Species-specific differences warrant careful consideration in experimental research. For example, transcriptomic data from murine multi-tissue macrophages (ImmGen) demonstrate robust expression of GPR65 across multiple macrophage subsets. In humans, tissue and immune cell distributions of GPR65 and GPR132, as reported by HPA, GTEx, and FANTOM5 datasets, exhibit greater tissue specificity and inter-individual variability, with GPR132 displaying relatively broad transcriptional signals across multiple tissues (70). Such interspecies differences may influence the extrapolation of findings from model organisms to clinical settings. At the level of structural motifs, the evolution of proton-sensing GPCRs involves not only receptor family diversification but also the conservation and fine-tuning of titratable residue networks. Wen et al. recently resolved the structures of mouse GPR4 and Xenopus GPR4 under different pH conditions and showed that the optimal activation pH of GPR4 correlates with the physiological blood pH range of each species (71). In these structures, key histidine residues in ECL2 and the transmembrane region, including H^ECL2-45.47^ and H^7.36^, promote the formation of a polar interaction network between ECL2 and the seven-transmembrane core upon protonation, thereby defining a conserved activation route for GPR4 across species (71). In Xenopus GPR4, an additional H^ECL2-45.41^ site is associated with a more acidic optimal activation pH, suggesting that the acquisition or repositioning of a limited number of proton-sensitive residues can tune the acid-sensitivity threshold of the receptor (71). Similarly, proton sensing by GPR68 is not mediated by a single proton-binding site but depends on a distributed network of titratable residues extending from the extracellular region to the transmembrane core; specific histidine residues within this network may influence both the pH response range and G protein-coupling bias of the receptor (72). These findings suggest that the evolutionary adaptation of proton-sensing GPCRs is achieved through a balance between conserved histidine/acidic residue networks that preserve core acid-sensing capacity and receptor- or species-specific residue configurations that adjust sensitivity to distinct pH niches, such as blood, mucosa, tumors, and inflamed tissues. Genetic variation or altered expression of these conserved motifs may therefore modify acid-sensing thresholds and potentially influence vascular permeability, inflammatory amplification, and susceptibility to diseases associated with acidic microenvironments. Structural biology approaches, including cryo-electron microscopy and molecular dynamics simulations, provide additional support for cross-species comparisons, enabling more precise identification of proton-binding sites and transmembrane structural variations. Therefore, differences in sensor expression profiles between experimental models and humans must be carefully considered during clinical translation.

Overall, the pH-sensing network encompasses multiple layers, including membrane receptors, ion channels, and intracellular factors. These molecules, characterized by distinct structural features, activation pathways, and distribution patterns, collectively form the molecular basis for cellular detection of acid–base fluctuations. Their coordinated presence enables cells to efficiently capture spatiotemporal changes in microenvironmental pH and convert this physicochemical signal into downstream biological effects, thereby providing a foundational framework for inflammatory regulation and homeostatic maintenance.

## 3 pH-dependent inflammatory regulatory networks

The widespread presence of acidic microenvironments in inflammatory settings positions pH sensors as central nodes within signaling networks. These molecules convert fluctuations in extracellular proton concentrations into intracellular signals through distinct activation mechanisms, which are subsequently amplified across transcriptional, metabolic, and proteostatic layers of cellular reprogramming. Although different pH sensors exhibit unique structural features and modes of activation, they ultimately converge on core inflammatory signaling pathways, forming regulatory systems that are partially overlapping yet functionally distinct.

### 3.1 Signal transduction

Proton-sensing GPCRs represent major membrane-level entry points for transducing extracellular acidification into intracellular signaling. Early studies largely attributed their activation to the protonation of extracellular histidine residues. However, recent structural and functional studies of this receptor family suggest that proton sensing by GPR4, GPR65, and GPR68 is not governed by a single histidine switch, but instead involves cooperative networks of titratable residues distributed across extracellular loops, the extracellular vestibule, and the transmembrane core. In addition to histidine residues, titratable carboxylate residues such as aspartate and glutamate can participate in pH-dependent rearrangements of hydrogen bonds, salt bridges, and polar interactions. These changes couple extracellular acidification to conformational movements of TM3 and TM6 and to canonical GPCR activation motifs, ultimately reshaping the intracellular G protein-binding interface (72). At the level of signal coupling, GPR4 and GPR65 primarily increase cAMP levels through Gs proteins, whereas GPR65 may also engage G12/13–RhoA signaling to regulate cytoskeletal remodeling and cell migration in specific cellular contexts (27, 32–34). GPR68 (OGR1) has traditionally been viewed as a mainly Gq–PLC–IP3–Ca²^+^-coupled receptor. Recent cryo-EM structures, however, indicate that interactions involving TM5/TM6, ICL2/ICL3, helix 8, and the C-terminal α5 helix of Gα can support both Gs and Gq coupling (73). Such dual coupling may reflect the propagation of acidification-induced rearrangements from extracellular histidine/carboxylate residue networks to the intracellular receptor interface, thereby giving rise to pH-, cell type-, and G protein expression-dependent signaling bias. Thus, the inflammatory functions of proton-sensing GPCRs are best understood as a continuum linking titratable residue networks, receptor conformational rearrangement, and biased G protein coupling, rather than as a simple histidine-protonation model.

Unlike receptor-type GPCRs, ASICs and TRPs function as channel-type pH sensors, with their primary signaling feature being changes in transmembrane ion flux (Na^+^/Ca²^+^) (54, 74). ASICs rapidly open in response to sharp decreases in pH, predominantly mediating Na^+^ currents; under specific subtypes or conditions, Ca²^+^ permeability or intracellular Ca²^+^ elevation has been observed (e.g., in ASIC1a/ASIC3-related studies), leading to rapid alterations in membrane potential. ASIC activity exhibits interaction and feedback regulation with inflammatory pathways such as MAPK and NF-κB, in a manner dependent on cell type and stimulus context. A defining characteristic of ASIC signaling is its rapid, transient activation and propensity for desensitization, rendering it well suited as an “alarm” mechanism during acute acidification (74–77).

In contrast, TRP channels such as TRPV1 and TRPA1 can generate relatively sustained Ca²^+^ influx under acidic conditions (although Ca²^+^-dependent desensitization occurs, influenced by the magnitude and duration of pH change as well as cell type). Calcium, acting as a second messenger, can engage transcriptional and metabolic regulatory axes such as CaMK–CREB and NFAT (direct evidence supports the TRPV1–CaMK–CREB pathway) (78–80). The kinetic differences between ASICs and TRPs suggest that ASICs are more closely associated with acute inflammatory responses, whereas TRPs are more likely to exert sustained regulatory effects in chronically acidified environments.

### 3.2 Transcriptional reprogramming in acidic microenvironments

Following conversion by membrane sensors, acidic signals are rapidly transmitted to the transcriptional level, thereby shaping inflammatory gene expression programs. NF-κB is a central regulator within this network; however, its response to acidification is highly context dependent. Although enhanced phosphorylation and nuclear translocation of NF-κB have been observed under acidic conditions in models such as vascular endothelial cells, acidification can markedly suppress NF-κB activation in specific settings, including hypercapnic acidosis, thereby exerting anti-inflammatory effects (32, 81, 82). The MAPK cascade likewise exhibits sensitivity to acidification. Activation of the ERK, JNK, and p38 pathways promotes increased transcriptional activity of downstream factors such as AP-1 (83, 84). The impact of acidification on HIF-1α is context-dependent: in certain tumor settings, acidic conditions may enhance HIF function via HSP90-mediated pathways; however, in environments where acidification and hypoxia coexist, some studies have reported reduced stability of HIF-1α under acidic stress (59, 60). It should be noted that most evidence regarding these classical transcription factors derives from *in vitro* experiments and animal models, while validation in clinical samples remains limited. Beyond these canonical factors, epigenetic regulators provide an additional regulatory dimension. Recent studies demonstrate that acidification can remodel BRD4/MED1 transcriptional condensates and chromatin binding in a locus-specific manner, thereby modulating inflammatory gene expression and establishing a negative feedback mechanism termed “acid-sensitive epigenetic regulation” (22). Specifically, under acidic conditions, BRD4 dissociates from chromatin regions associated with certain pro-inflammatory genes, leading to decreased transcriptional output and exerting a braking effect on inflammatory responses. This finding highlights a negative feedback phenomenon of acid-induced epigenetic reprogramming and represents a notable recent advance in the field. Overall, GPCRs influence classical inflammatory transcription via cAMP/Ca²^+^ signaling axes; ASICs and TRPs trigger rapid and desensitizable activation of transcription factors through ion flux; whereas nuclear regulators such as BRD4 mediate locus-specific and relatively sustained epigenetic remodeling, in a manner dependent on cell type and the pattern of acidification.

### 3.3 Metabolic adaptation and its interaction with inflammation

Metabolic reprogramming represents another central component of the pH-dependent inflammatory network. Notably, metabolic alterations and pH fluctuations often reinforce one another, forming a self-amplifying loop. Local acidification frequently accompanies enhanced glycolysis, and the two processes establish a positive feedback relationship. The acid sensitivity of HIF-1α plays a pivotal role in this context. Multiple studies indicate that acidification can regulate HIF-1α through various mechanisms, commonly resulting in stabilization and a shift toward glycolytic metabolism, although reduced HIF-1α stability under specific hypoxic conditions has also been reported. GPCR pathways also contribute significantly to metabolic reprogramming. GPR65 primarily signals through Gs to elevate cAMP levels, and the interaction between cAMP and AMPK is context-dependent—AMPK may be inhibited via PKA or activated through the EPAC–CaMKKβ axis—thereby reshaping cellular energy metabolism (35, 85, 86). Sustained calcium signaling triggered by TRP channels can activate mitochondrial metabolic regulators, such as PGC-1α, thereby adjusting cellular energy supply to accommodate environmental stress (87). Lipid metabolism is likewise sensitive to pH changes. Under acidic conditions, the balance between fatty acid synthesis and oxidation shifts, with increased production of inflammation-associated lipid mediators (88). Amino acid metabolism is also altered in acidic environments: acidification enhances glutamine catabolism (releasing ammonia to buffer acidity) while suppressing the conversion of arginine to nitric oxide (89–91). Different pH sensors exert distinct influences on metabolic pathways. HIF-1α primarily drives glycolysis; GPCRs modulate lipid and amino acid metabolism through cAMP or Ca²^+^ signaling; ASICs and TRPs more prominently affect mitochondrial function via ion homeostasis and calcium dynamics. Metabolic alterations, in turn, exacerbate acidification, forming a self-amplifying “acidification–metabolism–inflammation” circuit. Within this loop, pH transporters (such as NHE1 and MCTs), HIF, cPLA_2_/COX-2, and AMPK represent potential therapeutic intervention targets. Collectively, these metabolic adaptations demonstrate that pH fluctuations not only modulate signal transduction but also determine the trajectory of inflammatory responses at the level of cellular energy metabolism.

### 3.4 pH-dependent regulation of proteostasis

The impact of acidification on protein homeostasis adds a more concealed layer to inflammatory networks. Mild acidification can alter protein folding states, thereby activating molecular chaperone systems to maintain functional proteomes (92). Proteasomal and autophagic pathways are significantly modulated under acidic conditions. For example, under mild to moderate acidification, 26S proteasome activity tends to decline, which is associated with the accumulation of substrates and signaling proteins and may accelerate the buildup of inflammation-related transcription factors (93). In parallel, defective lysosomal acidification can impair autophagic flux by reducing autolysosomal degradative capacity and/or disrupting autophagosome–lysosome fusion, thereby compromising the clearance of inflammasome-related or other inflammatory cargoes (94, 95).

In addition, post-translational modifications are highly sensitive to acid–base status. Through proton-sensing GPCRs and calcium signaling, acidification couples extracellular pH fluctuations to kinase and deacetylase networks. Notably, distinct G-protein signaling pathways exert differential effects on the subcellular localization of histone deacetylase 5 (HDAC5): the Gαq–PKD/PKCμ axis can promote the nuclear export of HDAC5, thereby relieving transcriptional repression, whereas the cAMP–PKA pathway may inhibit its nuclear export under specific conditions. This fine-tuned regulation of HDAC5 shuttling, together with the bidirectional coupling between intracellular pH and histone acetylation status, collectively rewires inflammatory transcriptional programs (95–97).

Distinct pH sensors contribute differently at the level of proteostasis. GPCRs and TRPs indirectly influence the activity of modifying enzymes through signaling cascades; ASICs, via abrupt ion influx, can induce ER stress and activation of the unfolded protein response (UPR), accompanied by perturbations in protein homeostasis; nuclear factors such as BRD4 directly display pH-dependent alterations in chromatin-binding profiles, functioning as “sensors” within proteostatic regulation (22, 98).

## 4 Roles of pH sensors in physiological and pathological contexts

Inflammation is a fundamental defense process that enables the host to eliminate pathogens and repair tissue injury, and acidic microenvironments are present throughout nearly all stages of the inflammatory response. As direct detectors of acid–base fluctuations, pH sensors convert proton signals into intracellular molecular responses, thereby shaping the nature, magnitude, and duration of inflammation. Under homeostatic conditions, these sensors help maintain immune sensitivity while preventing excessive activation. In contrast, under pathological conditions, acidified environments drive aberrant activation or reprogramming of pH sensor expression profiles, thereby sustaining inflammation and exacerbating tissue damage.

### 4.1 Physiological immune homeostasis

In physiological settings, pH sensors contribute to maintaining a low inflammatory baseline and promoting timely resolution by modulating activation thresholds, signal duration, and negative feedback mechanisms in endothelial and immune cells. For example, in lymphocytes, GPR65 can be modestly activated under mildly acidic conditions, increasing intracellular cAMP via the Gs–adenylate cyclase pathway. Through downstream axes such as PKA/CREB–IκB, this signaling elevates the effective activation threshold of T cells and prevents unnecessary activation (25, 99).

In barrier tissues such as the intestine and skin, GPR4, TRPV1, and TRPA1 are sensitive to mild local acidification and can generate transient or low-amplitude signals in a context-dependent temporal manner. These signals modulate epithelial–immune cell crosstalk and help restrain excessive inflammatory expansion (55, 56, 100, 101). The “rapid activation–readily desensitizing” kinetics of ASICs allow them to produce transient Na^+^ currents (accompanied by context-dependent Ca²^+^ influx) during early acidification, serving as early warning signals that connect to subsequent stress and inflammatory pathways (74, 75, 77). Such low-level activation events do not directly provoke inflammatory storms but instead function as “sentinel” mechanisms within inflammatory homeostasis. Overall, these effects are characterized by low amplitude and short duration, suggesting that pH sensors under homeostatic conditions primarily act to limit excessive inflammatory responses. It should be noted that current understanding of these mechanisms is largely derived from animal studies, with limited direct clinical validation.

### 4.2 Driving pathological inflammatory imbalance

In chronic inflammatory diseases, the function of proton-sensing GPCRs exhibits marked tissue dependence. Comparative analyses of rheumatoid arthritis (RA) and inflammatory bowel disease (IBD) indicate that the same receptor family may either contribute to maintaining anti-inflammatory homeostasis or, when aberrantly upregulated, become a molecular driver of persistent inflammation in distinct microenvironments (**Table 1**). Notably, findings regarding GPR65 expression changes in RA are inconsistent, potentially reflecting differences in sampling sites, patient stratification, and detection methodologies.

**Table 1:** Modes by which pH sensors drive inflammatory dysregulation in distinct pathological microenvironments.

Pathophysiological microenvironment/representative diseases	Acidification patterns and time course	Key pH sensors	Primary downstream pathways/effectors	Major pathological consequences	References
Chronic Inflammation: Rheumatoid Arthritis (RA) Synovium	Chronic/recurrent acidification (co-driven by local metabolism and inflammatory cell infiltration); predominantly “low-grade but persistent.”	GPCRs: GPR65 (TDAG8); Ion Channels: ASIC3	GPR65: Gs → cAMP/PKA (often involves immunosuppression or “threshold modulation,” though reported results may vary across layers); ASIC3: Acid-triggered Na^+^/Ca^2+^channels → altered excitability of neurons/synoviocytes and inflammatory amplification.	Pain/hyperalgesia; synoviocyte inflammatory response; potential inconsistencies in GPR65 expression changes across different studies.	(102, 103)
Chronic Inflammation: Inflammatory Bowel Disease (IBD) Mucosa and Interstitium	Chronic acidification (barrier disruption + inflammatory cell infiltration + tissue metabolic remodeling); functions as a persistent “microenvironmental background variable.”	GPCRs: GPR4; GPR68 (OGR1); GPR65	GPR4: Gs → cAMP/EPAC; potentially linked to NF-κB-related inflammatory gene programs; GPR68: Ca^2+^ signaling and inflammatory/fibrotic pathways; both are more closely associated with “inflammatory maintenance and fibrotic progression.”	Maintenance of mucosal inflammation; alterations in permeability; specific pathways linked to fibrosis.	(101, 104–106)
Chronic Airway Acidification: Asthma/COPD	Fluctuations in airway surface acidification (rapid decline upon acute stimulus; remains low during chronic course); associated with cough/irritation reflexes.	TRP Channels: TRPV1/TRPA1; ASICs (airway sensory neurons/vagal pathways)	TRPV1/TRPA1: Multimodal (acid/heat/irritants) → Cainflux → neuropeptide release and neurogenic inflammation; ASICs: Rapid depolarization → acute reflexes (e.g., cough).	Acute stimulus reflexes (cough/bronchoconstriction); possible coexistence of chronic hyperresponsiveness and hyperalgesia.	(107–110)
Acute Severe Acidification: Ischemia/Reperfusion (I/R) Injury	Acute, high-magnitude, and transient acidification (ischemic/reperfusion phases), prone to creating an “acidotoxicity window.”	ASICs: ASIC1a, ASIC3; TRP: TRPA1/TRPV1	ASICs: H^+^-gated channels → robust ionic flux → Ca^2+^ overload/cellular damage → inflammatory burst; distinct from the “signal-modulating” role of GPCRs in chronic inflammation, acting more as a “triggering” mechanism.	Acute cellular injury; inflammatory burst; TRPA1/TRPV1: involved in pain, inflammation, and reperfusion responses in specific tissues/models.	(6, 111)

In respiratory diseases, acidification shapes the temporal characteristics of inflammation through coordinated activation of multiple ion channels. TRP channels and ASICs display complementary functions under acidic stimulation: TRPs tend to sustain chronic inflammation and neurogenic amplification, whereas ASICs are highly sensitive to acute acidification and more readily trigger rapid reflexive responses. This functional division may explain why airway acidification is associated with both exacerbation of chronic inflammation and acute symptomatic attacks.

Distinct from the GPCR-dominant regulatory pattern observed in chronic inflammation, ischemia–reperfusion injury exemplifies an acute inflammatory amplification mechanism mediated by acid-sensing ion channels. Intense and transient ion flux acts as a direct trigger of cellular injury and inflammatory burst, conferring prominent pathogenic significance to ASIC signaling in this context (**Table 1**).

The tumor microenvironment represents another distinctive inflammatory state. In chronically acidified tumors, proton-sensing GPCRs (notably GPR68 and GPR132) undergo sustained activation, primarily reinforcing immunosuppressive and pro-inflammatory programs in tumor-associated myeloid cells (TAMs/MDSCs), thereby maintaining chronic inflammation and promoting tumor immune evasion. Under acidic conditions, these cells secrete high levels of inflammatory and immunosuppressive mediators (such as IL-6, TNF, PGE_2_, TGF-β, lactate, and others), which both sustain local chronic inflammation and create permissive conditions for immune escape (37, 38, 112).

pH-dependent regulation by the nuclear factor BRD4 further highlights the long-term coupling between acidification and inflammation. Under acidic conditions, BRD4 condensates and chromatin-binding profiles undergo locus-specific remodeling, thereby reconfiguring gene expression landscapes related to inflammation and immunoregulation (often in a negative-feedback direction) (22). In persistently acidified tumors, these findings suggest that BRD4 may function as an endogenous nuclear sensor of acidic stress and contribute to context-dependent epigenetic remodeling of inflammatory and immunoregulatory gene programs. Rather than indicating a uniform suppression of anti-tumor immunity, current evidence more strongly supports a locus-specific and cell-type-dependent reorganization of transcriptional states, with potential implications for macrophage-associated immunoregulation within the tumor microenvironment. This mechanism underscores that pH sensors not only contribute to the initiation of inflammatory signaling but can also determine the trajectory toward chronicity through transcriptional reprogramming. Unlike inflammatory diseases such as RA and IBD, acidification in the tumor microenvironment is more sustained, and pH sensors therefore tend to drive long-term immunoregulation.

In chronically acidified tumors, pH-dependent regulation by BRD4 may provide an important link between microenvironmental acidification and immune reprogramming. Recent work has shown that acidic pH can disrupt BRD4-containing transcriptional condensates and remodel inflammatory gene expression in macrophages, supporting the view that BRD4 can function as an intracellular sensor of acidic stress. Independently, BRD4/BET activity has also been implicated in the maintenance of immunoregulatory macrophage states and dysfunctional CD8^+^ T-cell programs in tumor and leukemia models. These observations raise the possibility that acidity-driven BRD4 remodeling may intersect with tumor-associated macrophage polarization and CD8^+^ T-cell dysfunction in the tumor microenvironment. However, direct evidence for a fully established acidic pH–BRD4–TAM/CD8 causal axis remains limited. At present, this connection is better interpreted as a biologically plausible mechanistic bridge rather than a conclusively demonstrated pathway (22, 113–116).

In recent years, acidification-induced remodeling of cell-surface glycans and the glycocalyx has emerged as a mechanism linking pH regulation, metabolic adaptation, and the tumor immune microenvironment. Cell-surface glycosylation is not a static feature, but can undergo rapid and reversible remodeling in response to growth factor stimulation or acidic stress. For example, acidification-associated desialylation can alter galectin binding to glycosylated lipids and proteins, thereby affecting the endocytosis and recycling of membrane proteins such as integrins, with consequences for cell migration (117).

Moreover, tumor acidosis can remodel glycocalyx architecture to regulate lipid uptake and ferroptosis sensitivity, suggesting that glycan networks may contribute to metabolic plasticity in acidic microenvironments (118). Given that glycosylation and glycocalyx organization can influence immune checkpoint stability, cell adhesion, receptor clustering, and immune-cell recognition, acidification-induced glycan remodeling may represent an additional regulatory layer linking tumor metabolic adaptation, immune evasion, and therapy response. Future studies integrating glycomics, spatial transcriptomics, and pH imaging may help define the pathological significance of the “pH–glycocalyx–immune regulation” axis in acidic microenvironments.

**Figure 3:**
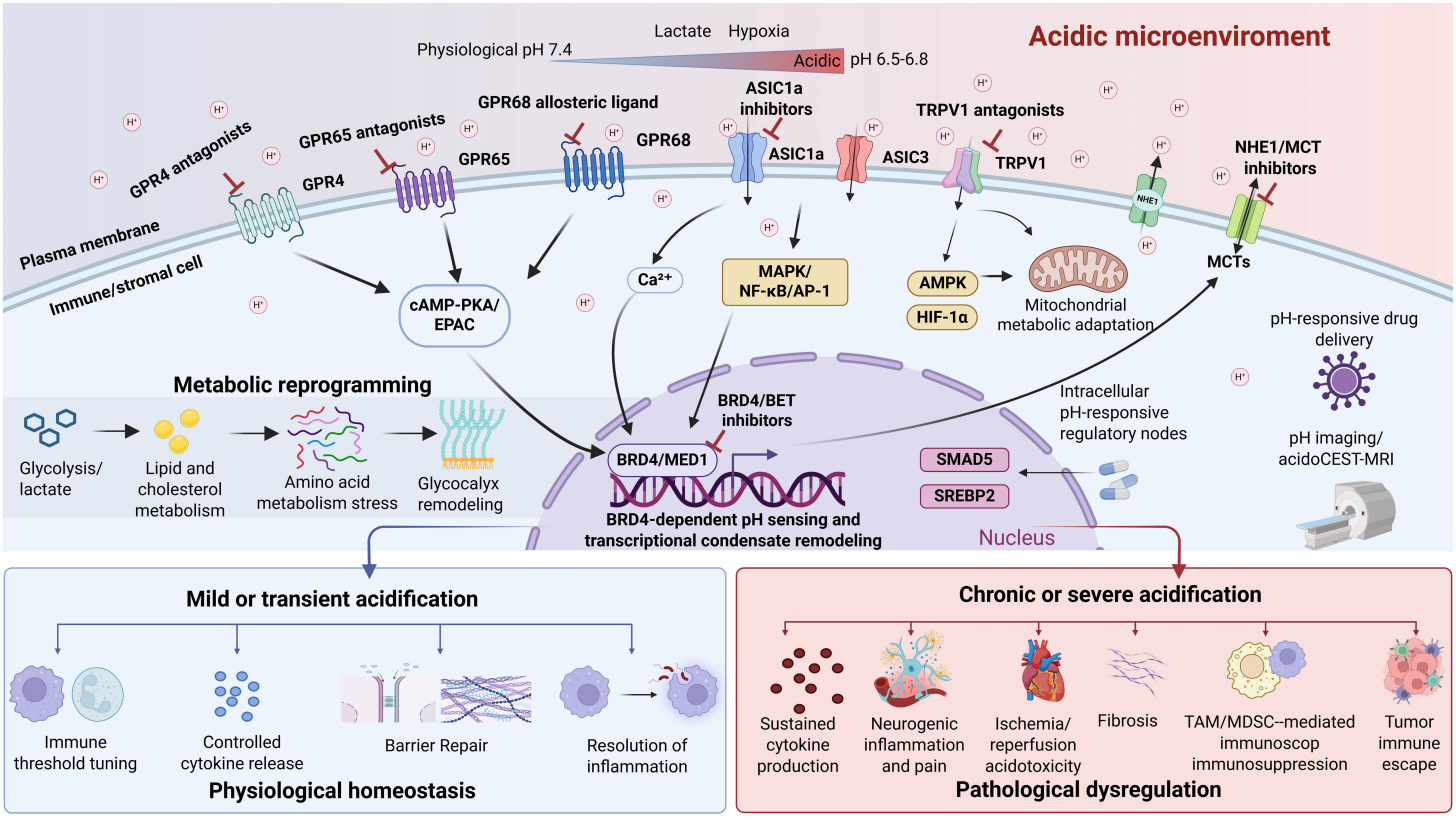
From acidic microenvironments to immune reprogramming: pH sensing in homeostasis, disease, and therapy.

Distinct classes of pH sensors exhibit contrasting roles in inflammation. GPCRs tend to function as “amplifiers” in chronic inflammation by sustained activation and upregulation; ASICs act as “triggers” of acute inflammation, responding rapidly to abrupt acidification and driving tissue injury; TRPs integrate inflammatory and nociceptive signaling, tightly coupling inflammatory responses with symptomatic sensation; and nuclear factors such as BRD4 and HIF-1α provide epigenetic and metabolic foundations for long-term inflammatory maintenance. Together, the complementary actions of these molecules across different temporal scales and tissue contexts shape the dynamic landscape of inflammation under acidic conditions (**Figure 3**).

## 5 Clinical translation and therapeutic potential

As key molecular mediators of cellular acid–base sensing, pH sensors have increasingly attracted attention in the field of clinical translation. Beyond serving as conceptual tools in basic research, they are emerging as promising targets for diagnosis and therapy. With advances in molecular biology and imaging technologies, the clinical relevance of pH sensors is becoming progressively evident, particularly across three major domains: diagnostics, therapeutic intervention, and precision medicine.

### 5.1 pH sensors as diagnostic beacons and biomarkers

In clinical disease diagnosis, the expression patterns and activation states of pH sensors are closely associated with disease progression. Clinical omics analyses and patient-derived sample studies indicate that dysregulation of pH sensors correlates with disease activity. For example, acidification is commonly observed in the joint microenvironment of rheumatoid arthritis (RA), and GPR65 in synovial fibroblast-like cells can be activated under acidic conditions to promote the release of inflammatory mediators. This suggests that GPR65 may serve as a biomarker reflecting acidic microenvironmental signaling for disease assessment. However, different studies have reported inconsistent findings regarding whether GPR65 expression is upregulated or downregulated in RA synovial tissues, and the quantitative relationship between GPR65 expression, local pH levels, and inflammatory persistence remains unresolved, requiring further prospective investigation (94, 103).

In patients with inflammatory bowel disease (IBD), increased expression of GPR4 and GPR68 has been detected in intestinal mucosal immune cells, and this upregulation correlates with clinical disease activity indices (100, 101, 106), supporting their potential utility as molecular indicators of disease severity.

Animal model studies have also provided preliminary evidence regarding the role of pH sensors. For instance, changes in ASIC channel activity and the impact of GPR65 deficiency on inflammatory responses have been observed in experimental models, although these findings should be cautiously extrapolated to human clinical settings. Clinical trial evidence is also beginning to emerge. In patients with asthma and chronic obstructive pulmonary disease (COPD), TRPV1 exhibits heightened sensitivity and is associated with cough hyperresponsiveness. Antagonists targeting TRPV1 have shown the ability to reduce stimulus-induced cough reflex sensitivity in early-phase clinical trials, although their effects on major clinical endpoints remain uncertain. This suggests that TRPV1 may currently be more suitable as a biomarker or patient stratification indicator rather than a direct therapeutic target (119, 120).

In tumor and inflammatory microenvironments characterized by acidification, BRD4 acts as a nuclear pH sensor that reprograms transcriptional condensates and chromatin accessibility, consistent with remodeling of gene-expression programs linked to inflammation and immunoregulation (22, 121). These epigenetic alterations provide new insights for molecular diagnostics in cancer. With the continued advancement of molecular detection platforms, incorporating pH sensors into molecular diagnostic frameworks for inflammatory diseases and tumors is becoming increasingly feasible. Therefore, pH sensors hold promise as potential biomarkers, although further prospective clinical studies are required to validate their diagnostic value.

### 5.2 pH sensing enables visualization of tissue acidity

Advances in imaging technologies have provided new avenues for realizing the diagnostic potential of pH sensors. Functional probes for detecting tissue pH have already been developed for magnetic resonance imaging (MRI) and positron emission tomography (PET). For example, probes based on peptides or small molecules can alter their signal intensity under acidic conditions, enabling quasi–real-time or dynamic imaging of tissue pH. If these technologies are combined with antibodies or ligands specific to pH sensors, it may become possible in the future to achieve dual *in vivo* visualization of both the “sensor” and the “acidification state,” thereby enabling more precise classification and localization of pathological lesions. Fluorescence imaging and nanoprobe-based approaches are also under investigation. For instance, high-resolution imaging strategies combining “pH-sensitive fluorophores + receptor ligands” have been demonstrated in ex vivo tissue sections, although these methods are currently limited to research applications (122–124). Notably, some advanced technologies are beginning to approach clinical translation. For example, acidoCEST-MRI (chemical exchange saturation transfer MRI) has been used in preliminary clinical studies to monitor tissue acidity and has demonstrated promising potential (125). Nevertheless, most of these imaging techniques remain at the research stage, and their routine application in clinical diagnosis is still under exploration.

### 5.3 Targeting pH sensors opens new avenues for precision medicine

At present, intervention strategies targeting pH-sensing-related pathways have begun to move from mechanistic studies toward therapeutic development, involving multiple levels, including proton-sensing GPCRs, acid-sensing ion channels, and nuclear pH-responsive regulatory factors (**Table 2**). The translational maturity of these targets varies substantially. Proton-sensing GPCRs are considered a class of targets with relatively high druggability because of their clear membrane localization and ligand-binding pockets; acid-sensing ion channels are more suitable for interventions in acute acidification injury, pain, or symptomatic responses, and their long-term disease-modifying effects still require further validation; whereas BET proteins represented by BRD4 suggest that acidic microenvironments may also affect inflammatory transcriptional programs at the epigenetic level, providing a new entry point for precision therapy (**Table 2**).

**Table 2:** Therapeutic strategies targeting pH-sensing pathways.

Strategy level	Target/strategy	Representative interventions	Major indications or models	References
Membrane receptors: proton-sensing GPCRs	GPR4	Small-molecule antagonists; representative candidates include NE-52-QQ57 and other GPR4 antagonists	Vascular inflammation, inflammation- and pain-related models; hyperinflammatory infection models, such as SARS-CoV-2-infected K18-hACE2 mice	(127, 128)
	GPR68 (OGR1)	Allosteric ligands/tool compounds, such as Ogerin and its derivatives; modulation of proton-induced GPR68 signaling	Inflammation, fibrosis, mucosal inflammation, and immunomodulation-related models	(23, 128)
	GPR65 (TDAG8)	Proton-dependent receptor modulation; potential small-molecule modulators or genetic/pharmacological interventions	Inflammatory bowel disease, immune-cell functional regulation, and tumor immune microenvironment	
Ion channels: acid-sensing ion channels	ASIC1a/ASIC3	Peptide inhibitors, such as PcTx1 and Hi1a; non-specific small-molecule blockers, such as amiloride and benzamil	Ischemic brain injury, myocardial ischemia/reperfusion injury, pain, and acute acidotoxic injury models	(42, 43, 111)
TRP channels: multimodal acid sensors	TRPV1	Small-molecule antagonists, such as XEN-D0501 and SB-705498	Chronic cough, airway hyperresponsiveness, and clinical or preclinical exploration of hypersensitivity-related syndromes	(120, 129)
Intranuclear/epigenetic pH-dependent regulatory factors	BRD4/BET family	BET inhibitors; representative agents include Pelabresib (CPI-0610) and other BET inhibitors	Myelofibrosis, hematologic malignancies, solid tumors, and remodeling of inflammatory transcriptional programs; Pelabresib combined with ruxolitinib has entered phase III clinical investigation for myelofibrosis	(126, 130–132)
pH-triggered delivery/release systems	pH-responsive liposomes, polymers, prodrug linkers, and tumor microenvironment-responsive platforms	Low-pH-triggered drug release; acid-sensitive liposomes, pH-responsive polymers, and acid-labile prodrug linkers	Site-specific release in acidic tumor or inflammatory microenvironments; when combined with receptor targeting or sensor-guided localization, these systems may enable “dual-precision” delivery	(133–135)

Several candidate interventions provide preliminary support for this therapeutic rationale. The BRD4/BET inhibitor Pelabresib (CPI-0610) has shown relatively clear clinical application potential in myelofibrosis, and its combination with ruxolitinib not only improves spleen response and symptom burden, but also suggests, in exploratory analyses, further improvement in pro-inflammatory cytokine levels and bone marrow fibrosis morphology compared with the control group, supporting the possibility that BET inhibitors may affect the disease microenvironment by regulating inflammatory transcriptional programs (126). At the membrane receptor level, the GPR4 antagonist NE-52-QQ57 has shown representative preclinical value in hyperinflammatory models, for example by improving survival in SARS-CoV-2-infected K18-hACE2 mice and reducing pulmonary inflammatory factors, chemokines, and viral load, suggesting that GPR4 may become a druggable target in inflammatory acidic microenvironments (127). At the ion channel level, the ASIC1a inhibitors PcTx1 and Hi1a provide relatively direct pharmacological evidence for acute acidification injury. Among them, PcTx1 shows neuroprotective effects in ischemic brain injury models, and Hi1a, as a potent double-knot peptide ASIC1a inhibitor, also shows tissue-protective potential in stroke models and myocardial ischemia/reperfusion injury models (43, 111). These studies indicate that the value of pH sensor-targeted therapy is not limited to a single disease type, but should be distinguished according to different pathological windows, such as chronic inflammatory transcriptional remodeling, endothelial inflammatory amplification, pain/neurogenic inflammation, and acute acidotoxic injury. It should be noted that most current intervention strategies still cannot achieve strict selective activity under acidic pH conditions *in vivo*, and their therapeutic relevance still depends on the specific disease context, drug delivery efficiency, and validation of target engagement.

Beyond directly targeting sensors, the development of acid-sensitive drug delivery systems has provided innovative therapeutic strategies. Nanocarriers based on polymers or liposomes can undergo structural disassembly under low pH conditions, enabling site-specific drug release. For example, some carefully designed prodrug systems link chemotherapeutic agents to pH-sensitive bonds, allowing active drugs to be released selectively within the acidic tumor microenvironment, thereby reducing systemic toxicity. Similarly, certain acid-responsive liposomal nanocarrier systems have demonstrated enhanced targeted drug release in tumor-bearing mouse models (133–135). If these systems can be combined with the specific localization properties of pH sensors, future strategies may achieve dual precision drug delivery through both “sensor-mediated targeting” and “acid-triggered release,” significantly improving drug utilization efficiency. However, ensuring the acid-selective responsiveness of these delivery systems within the complex *in vivo* environment remains a major challenge.

Personalized medicine has also opened new avenues for pH sensor research. Significant heterogeneity exists among patients in both the degree of tissue acidification and the expression profiles of pH sensors, presenting both challenges and opportunities for clinical intervention. The application of single-cell RNA sequencing and spatial transcriptomics has revealed cell type–specific differences in sensitivity to acidification signals. For instance, GPR132 (responsive to lactate/acidification) and GPR68 are frequently upregulated in tumor-associated macrophages (TAMs), whereas dominant expression of GPR4 appears to be more dependent on tissue context (such as tumor vascular endothelium) and patient background (38, 135, 136). These differences suggest that future therapeutic strategies may need to stratify patients based on their sensor expression profiles in order to select the most appropriate pharmacological interventions. In addition, integration of metabolomics with imaging-based analyses will further help delineate patient-specific “acidification landscapes,” providing an important foundation for precision medicine.

## 6 Conclusion and discussion

Recent studies have gradually reshaped our traditional understanding of the role of pH in inflammatory microenvironments. pH is no longer regarded merely as a metabolic byproduct within inflamed tissues but is increasingly recognized as a key signal capable of actively regulating immune responses. Acidic microenvironments are widely present in infection, ischemia, chronic inflammation, and tumors. Immune cells rely on multiple classes of pH sensors to convert extracellular acidification signals into finely tuned molecular responses. Membrane-level proton-sensing GPCRs, ASICs, and TRPs can rapidly activate classical inflammatory pathways in response to external stimuli, whereas nuclear factors such as HIF-1α and BRD4 integrate acid–base fluctuations into transcriptional and epigenetic regulatory networks. In particular, studies on BRD4 as a noncanonical pH sensor demonstrate that acidic conditions can reshape transcriptional condensates and chromatin-binding landscapes, thereby determining the expression patterns of inflammatory genes. Together, these advances establish the “pH–sensor–inflammation” axis as a new regulatory framework for understanding immune responses and disease pathogenesis.

From the perspectives of temporal dynamics, spatial localization, and signaling mechanisms, different classes of proton sensors fulfill complementary roles in inflammatory regulation. Membrane receptors and channels, including GPCRs, ASICs, and TRPs, are localized at the plasma membrane and can detect and respond to local acidity changes within seconds to minutes, rapidly transmitting signals through second messenger pathways such as cAMP and Ca²^+^ to regulate inflammatory mediator release, cell migration, and other immediate responses. In contrast, nuclear sensing factors such as BRD4 perceive pH changes within the nucleus and primarily reshape inflammatory responses over longer time scales—hours or longer—by modulating transcriptional and epigenetic programs. In other words, membrane sensors translate acidic signals into rapid immune activation and nociceptive reflexes, whereas BRD4 governs sustained transcriptional reprogramming of inflammatory genes, providing a molecular basis for the long-term maintenance and memory of inflammatory states. The coordinated division of labor across temporal and spatial scales enables the host to respond rapidly to acute acid stress while simultaneously adapting to chronic acidified environments, thereby forming a dynamic and integrated inflammatory response system.

Research centered on this axis has also opened new avenues for clinical application. Small-molecule modulators targeting proton-sensing GPCRs, selective inhibitors of ASICs and TRPs, and BET inhibitors acting on nuclear regulators such as BRD4 have demonstrated therapeutic potential in models of inflammatory diseases and cancer and are gradually entering early-phase clinical exploration. Given that BRD4 activity exhibits pH dependence, such inhibitors may exert enhanced selectivity within acidic lesions, potentially offering advantages for the precision treatment of refractory inflammatory diseases and tumors. The emergence of acid-responsive nanomedicine delivery platforms further provides new strategies for targeted therapy within inflammatory or tumor microenvironments. These developments suggest that pH is not merely a disease-associated marker but may also represent an important entry point for future immune interventions.

Despite these advances, many critical questions remain unresolved. Protein condensates and phase separation processes may represent underexplored mechanisms of pH sensing, as acidification-induced alterations in weak molecular interactions could constitute an additional regulatory pathway influencing gene transcription and cell fate decisions. Furthermore, accurate spatiotemporal measurement of pH *in vivo* remains a major challenge. Existing techniques often fail to capture the complex and heterogeneous acid–base gradients within inflamed tissues, and high-resolution, real-time imaging approaches will be essential for elucidating the spatial and temporal dynamics of pH signaling. Another layer of complexity arises from the multiple stresses present in inflammatory microenvironments. Acidification frequently coexists with hypoxia, reactive oxygen species accumulation, and nutrient deprivation, yet how cells integrate these signals into coherent regulatory decisions remains poorly understood.

Cell-type–specific differences among immune populations also warrant further investigation. To date, most studies of pH-dependent transcriptional regulation have focused on macrophages. Whether similar sensing and response mechanisms exist in T cells, dendritic cells, neutrophils, and natural killer (NK) cells remains unclear. For example, it is not yet known whether acidification alters T cell receptor activation thresholds, modulates the balance between effector and regulatory T cell subsets, or influences antigen presentation and migration in dendritic cells. Some gene regulatory processes also appear to exhibit pH dependence; for instance, the expression of Ifnb1 has been linked to IRF2 degradation under certain conditions, although the molecular mechanisms remain to be fully elucidated.

Interspecies differences further complicate clinical translation. Mice and humans differ in the magnitude and kinetics of tissue acidification as well as in the expression profiles of pH sensors, which may partly explain discrepancies between animal models and clinical observations. Future research should therefore place greater emphasis on human-based studies and integrate single-cell transcriptomics with spatial multi-omics to construct individualized “acidification maps.” Such strategies will not only deepen our understanding of disease heterogeneity but also facilitate the development of personalized therapeutic approaches.

Overall, the concept that pH functions as an active signaling regulator in inflammatory processes is gaining increasing recognition. From basic mechanisms to clinical translation, accumulating research continues to reveal how acidic microenvironments influence the nature, intensity, and outcomes of immune responses. With the discovery of new pH sensing mechanisms, advances in imaging technologies and multi-omics methodologies, and deeper insights into interspecies differences, the “pH–sensor–inflammation” axis is poised to become an important frontier at the intersection of immunology and clinical medicine. This framework not only deepens our understanding of immune regulation but also opens new therapeutic opportunities for inflammatory diseases and cancer. Future research should particularly focus on integrating single-cell sequencing with spatial omics, further elucidating noncanonical pH sensing mechanisms such as those mediated by BRD4, and constructing individualized tissue acidification landscapes to accelerate the translation of this field from experimental models to clinical application.
